# Glucocorticoid Programing of the Mesopontine Cholinergic System

**DOI:** 10.3389/fendo.2013.00190

**Published:** 2013-12-13

**Authors:** Sónia Borges, Bárbara Coimbra, Carina Soares-Cunha, Ana P. Ventura-Silva, Luisa Pinto, Miguel M. Carvalho, José-Miguel Pêgo, Ana João Rodrigues, Nuno Sousa

**Affiliations:** ^1^Life and Health Sciences Research Institute (ICVS), School of Health Sciences, University of Minho, Braga, Portugal; ^2^ICVS/3B’s – PT Government Associate Laboratory, Braga/Guimarães, Portugal

**Keywords:** glucocorticoids, stress, acetylcholine, anxiety, fear, pedunculopontine tegmental nucleus, laterodorsal tegmental nucleus, ultrasonic vocalizations

## Abstract

Stress perception, response, adaptation, and coping strategies are individually distinct, and the sequel of stress and/or glucocorticoids (GCs) is also distinct between subjects. In the last years, it has become clear that early life stress is a powerful modulator of neuroendocrine stress-responsive circuits, programing intrinsic susceptibility to stress, and potentiating the appearance of stress-related disorders such as depression, anxiety, and addiction. Herein we were interested in understanding how early life experiences reset the normal processing of negative stimuli, leading to emotional dysfunction. Animals prenatally exposed to GCs (*in utero* glucocorticoid exposure, iuGC) present hyperanxiety, increased fear behavior, and hyper-reactivity to negative stimuli. In parallel, we found a remarkable increase in the number of aversive 22 kHz ultrasonic vocalizations in response to an aversive cue. Considering the suggested role of the mesopontine tegmentum cholinergic pathway, arising from the laterodorsal tegmental nucleus (LDT) and pedunculopontine tegmental nucleus (PPT), in the initiation of 22 kHz vocalizations and hypothetically in the control of emotional arousal and tone, we decided to evaluate the condition of this circuit in iuGC animals. Notably, in a basal situation, iuGC animals present increased choline acetyltransferase (ChAT) expression in the LDT and PPT, but not in other cholinergic nuclei, namely in the nucleus basalis of Meynert. In addition, and in accordance with the amplified response to an adverse stimulus of iuGC animals, we found marked changes in the cholinergic activation pattern of LDT and PPT regions. Altogether, our results suggest a specific cholinergic pathway programing by prenatal GC, and hint that this may be of relevance in setting individual stress vulnerability threshold.

## Introduction

Exposure to stressful events or synthetic glucocorticoids (GCs), such as dexamethasone, early in life, are a risk factors for the development of different neuropsychiatric disorders in adulthood, namely depression and anxiety (1). Such effects are partially mediated by de-regulation of the hypothalamic-pituitary-adrenal (HPA) axis, leading to altered GC secretion (2, 3), which can induce long-term molecular and functional changes in GC-sensitive nuclei. Importantly, several recent studies showed that high levels of GCs have a deleterious effect in the developing brain, inducing prominent neurochemical, structural, and molecular changes in several brain regions (4–8) culminating in the development of anxious and depressive-like traits (9–13).

GC programing effects in emotional behavior are far from being completely understood at a mechanistic level, but it has been shown that GCs modulate on the long-term the activity of particular neural pathways. In this perspective it is interesting to refer that early life stress/GC exert a powerful effect in the developing dopaminergic neurons, especially at the mesolimbic level (7). For example, we have shown that animals prenatally exposed to GC present marked hypodopaminergia and D2 epigenetic/expression changes, responsible for their anhedonia and motivational deficits, since administration of a dopamine precursor, l-DOPA, fully reverted the molecular and behavioral impairments (14, 15). Obviously, this dopaminergic de-regulation may also occur indirectly through modulation of upstream neurotransmitter systems. One promising candidate is the ascending cholinergic pathway, comprising projections from acetylcholine (ACh)-rich nuclei within the pons, particularly the laterodorsal (LDT) and pedunculopontine tegmental nuclei (PPT) to the ventral tegmental area (VTA) (16–18). In support of this, it was already shown that stress/GC strongly elicit cholinergic activity of these regions (19), and that they, in turn, critically affect basal and phasic activity of VTA neurons (20–23). Additionally, GC can putatively bind to GC-responsive elements of cholinergic players and control their expression, namely choline acetyltransferase (ChAT) (24) and acetylcholine esterase (AChE) (25), two enzymes of the cholinergic pathway. Also relevant is to note that the ascending cholinergic system is implicated in the control of the stress response by modulating hypothalamic pituitary adrenal (HPA) axis function (26–28) and in mediating the anxiogenic effects of stress (29–31).

Surprisingly, very few studies have focused on the impact of stress/GC exposure in the cholinergic system. Considering the lack of evidence, herein, we focused on the impact of prenatal exposure to GCs in the ascending cholinergic system, and the relevance of such changes in stress-related anxious and fear behaviors.

## Materials and Methods

### Animals and treatments

All manipulations were conducted in accordance with local regulations on animal care and experimentation (European Union Directive 2010/63/EU). Pregnant Wistar Han rats were subcutaneously injected with the synthetic GC dexamethasone at 1 mg kg^−1^ (*in utero* glucocorticoid exposure, iuGC animals), or with vehicle (CONT; control animals), on days 18 and 19 of gestation.

Dexamethasone dosage was selected based on our previous studies showing that this regimen effectively impairs HPA axis activity in a long-term basis (12). From a clinical perspective, guidelines on prenatal corticotherapy (32, 33) recommend single course administration (0.3–0.5 mg/kg), however, multiple courses of GCs are often administered (34), despite the lack of evidence of increased therapeutic efficacy. Nevertheless, we must consider the difficulty in the transposition of human doses to rodents due to ADME species specificity.

At weaning day, male offspring were house-paired randomly, according with prenatal treatment, under standard laboratory conditions: artificial 12 h light/dark cycle (lights on from 08:00 a.m. to 08:00 p.m.); room temperature 22°C; food and water were provided *ad libitum*. Animals derived from at least three different litters were used for all the experimental procedures.

### Behavioral tests

All tests were performed during the day period, except the confined cage and fear-conditioning protocols that were conducted during the night period (08:30 p.m. to 03:00 a.m.). All behavioral equipment was cleaned between animals (ethanol 10%) in order to remove any olfactory cues.

### Open field

The open field (OF) test was conducted in an arena (43.2 cm × 43.2 cm) with transparent acrylic walls and white floor (Med Associates Inc., St. Albans, VT, USA). Rats were placed in the center of the arena and movement was monitored over a period of 10 min with the aid of two 16-beam infrared arrays. Total distance traveled was used as an indicator of locomotor activity.

### Elevated plus maze

The elevated plus maze (EPM) test was carried out under bright white light. Animals were placed individually for 5 min in the center of a black polypropylene plus-shaped platform elevated 72.4 cm above the floor. The apparatus consisted of two open arms (50.8 cm × 10.2 cm) and two closed arms (50.8 cm × 10.2 cm × 40.6 cm) (MedAssociates Inc., St. Albans, VT, USA). The number of entries into each arm and the time spent therein were recorded.

### Light/dark box test

The light/dark box (L/D) test was performed inside the OF arena (43.2 cm × 43.2 cm) (MedAssociates Inc., St. Albans, VT, USA). A dark compartment was attached to one side with an opening facing the center of the arena. Animals were individually placed in the center of the illuminated part. The distance traveled and time spent in each compartment was recorded in a single trial of 10 min.

### Confined cage test

The confined cage test was performed in a non-restrictive Plexiglas cylinder (inner diameter 8.8 cm, length 22.2 cm), mounted on a Plexiglas platform and placed in a ventilated, sound-attenuated chamber (SR-LAB, San Diego Instruments, San Diego, CA, USA). A stainless steel grid was placed inside the cylinder, through which an electric current could be passed (shock chamber). A microphone and a video camera were placed inside the sound-attenuated chamber. The protocol was performed in two consecutive days, in which the animals were placed inside the shock chamber for 3 min. The ultrasonic vocalizations (USVs) and the percentage of total freezing time were measured.

### Fear-conditioning paradigm

The fear-conditioning test was performed in a non-restrictive Plexiglas cylinder (inner diameter 8.8 cm, length 22.2 cm), mounted on a Plexiglas platform and placed in a ventilated, sound-attenuated chamber (SR-LAB, San Diego Instruments, San Diego, CA, USA). The protocol was performed in three consecutive days (35). On the first day (habituation), each animal was placed in the shock chamber for 11 min. On the second day (conditioning), each subject was positioned inside the shock chamber for 3 min (no light, no shock). Afterwards, animals were exposed to six lights/shock pairings (0.4 ± 0.1 mA), with an inter-stimulus-interval (ISI) of 60 s. The shock was given for 500 ms, immediately after the cue light was turned off. On the following day (test day), after an initial phase of 3 min without light, animals were presented with a 20 s cue light for six times, but no shock was given (ISI of 60 s). During all procedures, USVs and freezing behavior were recorded.

### USVs analysis

An ultrasound microphone (CM16/CMPA, Avisoft Bioacoustics, Berlin, Germany) sensitive to frequencies of 10–200 kHz, placed 15 cm above the floor, was used in all experiments. The microphone was connected via an Avisoft UltrasoundGate 416H (Avisoft Bioacoustics) to a personal computer; USVs were recorded using the Avisoft-Recorder (version 5.1.04) with the following settings: sampling rate: 250,000; format: 16 bit. For acoustical analysis, recordings were transferred to Avisoft SASLab Pro (version 5.1.22, Avisoft Bioacoustics). This program was used in order to produce spectrograms of USVs by conducting a fast Fourier transformation (256 FFT-length, 100% frame, Hamming window filter, 50% time window overlap). These spectrograms had a frequency resolution ∼1.2 kHz and a temporal resolution ∼0.4 ms.

Twenty-two kilohertz call detection was provided by an automated threshold-based algorithm (threshold: −40 dB) and a hold time mechanism (hold time: 20 ms). A lower-cut-off-frequency of 18 kHz was used to reduce background noise.

Calls were also inspected manually to ensure that, when necessary, USVs not detected automatically could be subsequently included in the automatic parameter analysis.

### Immunohistochemistry

For immunohistochemistry (IHC), 11 rats were sacrificed by pentobarbital (Eutasil, Sanofi) anesthesia and transcardially perfused with 0.9% saline followed by 4% paraformaldehyde (pH 7.4 in 0.1 M phosphate buffer). Brains were removed and post-fixed for 48 h in 4% paraformaldehyde and then rinsed and stored in 30% of sucrose until sectioning. Brains were sectioned coronally, at a thickness of 50 μm, on a vibratome (VT1000S, Leica, Germany) and stored in cryoprotectant solution at −20°C until use.

Briefly, free-floating sections were pre-treated with 3% H_2_O_2_ in PBS, thoroughly rinsed in PBS, blocked with 2.5% fetal bovine serum (FBS) in PBS-Triton 0.3% for 2 h at room temperature, and then incubated overnight at 4°C with primary antibody goat anti-ChAT (Millipore, MA, USA; 1:1000). Afterwards, sections were washed, incubated with the secondary biotinylated anti-goat (Vector Lab., USA; 1:200) for 1 h, and processed with an avidin-biotin complex solution (ABC-Elite Vectastain reagent; Vector Lab, USA). Detection was done using 0.5 mg/ml 3,3′-diaminobenzidine. Sections were washed and mounted on glass slides, air-dried, counterstained with hematoxylin, and cover slipped with Entellan-New (Merck, Darmstadt, Germany).

### Immunofluorescence

Ninety minutes after completion of the fear-conditioning paradigm, 12 animals were deeply anesthetized with pentobarbital (Eutasil, Sanofi) and were transcardially perfused with 0.9% saline followed by 4% paraformaldehyde. Brains were removed and post-fixed in 4% paraformaldehyde. Coronal vibratome sections (50 μm) were first incubated with rabbit anti-c-fos primary antibody (1:1000; Ab-5, Calbiochem, USA), followed by incubation with goat anti-ChAT primary antibody (1:1000; anti-ChAT, Millipore, MA, USA). Secondary fluorescent antibodies were: Dylight 488-conjugated donkey anti-rabbit IgG (1:500, BioLegend), and Alexa Fluor 568-conjugated donkey anti-goat IgG (1:500, Invitrogen), respectively. Finally, all sections were stained with 4′,6-diamidino-2-phenylindole (DAPI; 1 mg/ml). For each animal, c-fos-positive cells within the PPT and LDT were analyzed after double staining with cholinergic (ChAT) marker and cell counts were performed by confocal microscopy (Olympus FluoViewTMFV1000, Hamburg, Germany). Estimation of cell density was obtained by crossing cell number values with the corresponding areas, determined using an Olympus BX51 optical microscope and the StereoInvestigator software (Microbrightfield, VT, USA).

### Stereological procedures

Eleven animals were perfused transcardially with 4% paraformaldehyde, under deep pentobarbital (Eutasil, Sanofi) anesthesia. Brains were included in glycolmethacrylate (Tecnovit 7100, Heraeus Kulzer, Wehrheim, Germany) and sectioned on a microtome as described in detail elsewhere (36). Every other 30 μm thick coronal section was collected on a gelatinized slide, stained with Giemsa, mounted with Entellan-New (Merck, Darmstadt, Germany), and cover slipped. Stereological procedures were performed by a blind observer.

Laterodorsal tegmental and PPT regions were outlined according to the atlas of Paxinos and Watson (37) and based on noticeable cytoarchitectural differences, namely density of cells and size of the perikarya.

Volume and neuronal number estimations were performed using StereoInvestigator software (Microbrightfield, VT, USA) and a camera attached to a motorized microscope (Axioplan 2, Carl Zeiss, Germany). The Cavalieri’s principle was used to assess volume. Briefly, every second section was used and the cross-sectional area was estimated by point counting (final magnification 112×). We used a test point system in which the interpoint distance, at the tissue level, was 150 μm for LDT and 200 μm for PPT. The volume of the region of interest was calculated from the number of points within its boundaries and the distance between sampled sections.

Average cell numbers were estimated using the optical fractionator method (38). Briefly, a grid of virtual 3D-boxes (LDT: 30 μm × 30 μm × 20 μm; PPT: 40 μm × 40 μm × 20 μm) equally spaced (using the same grid spacing as for volume estimations) was superimposed on every second section of the lamina of interest and cells within boxes were counted. Coefficients of error (CE) were automatically computed, according to the formulas of Gundersen et al. (39) for cell numbers and Gundersen et al. (40) for volume estimations. Glial cells were not included in the estimations, and the discrimination between neuronal and glial cell body profiles was based on the criteria described by Ling et al. (41) and Peinado et al. (42).

### Statistical analysis

Statistical analysis was performed in GraphPad Prism 5.0 (GraphPad Software, Inc., La Jolla, CA, USA). Statistical analysis between two groups was made using Student’s *t*-test or Mann–Whitney tests. Two-way analysis of variance (ANOVA) was used when appropriate. Bonferroni’s *post hoc* multiple comparison test was used for group differences determination. Non-parametric analysis (Mann–Whitney test) was used when normality of data was not assumed. Results are presented as mean ± SEM. Statistical significance was accepted for *p* ≤ 0.05.

## Results

### *In utero* glucocorticoid exposure impairs emotional behavior

Animals were exposed to a battery of behavioral tests that consisted of paradigms studying spontaneous exploratory behavior (OF), tasks of innate anxiety (EPM, L/D test, confined cage), and reactivity to adverse stimulus (version of fear-conditioning paradigm). Since USVs can give information on the emotional status of the animal, we decided to further complement the behavioral characterization by measuring USVs in these different paradigms.

**Figure 1:**
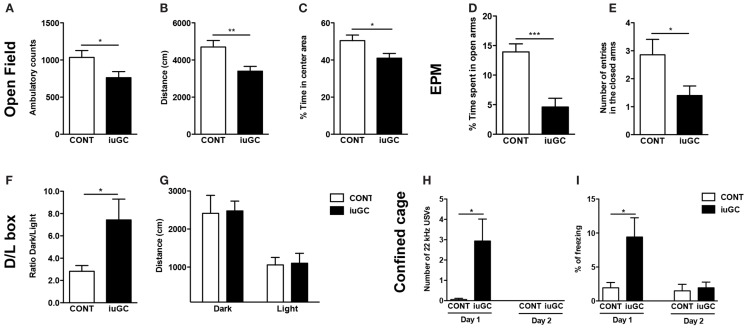
***In utero* glucocorticoid exposure induces an anxious-like behavior**. iuGC group presented a decrease in ambulatory counts **(A)**, total distance traveled **(B)**, and percentage of time in the center of the arena **(C)**. In the EPM, iuGC animals exhibited a decrease in the percentage of time spent **(D)** and number of entries **(E)** in the open arms of the maze, when compared with control group. **(F)** In the L/D test, iuGC presented an increase in the ratio dark/light, with no differences on the distance traveled in both compartments **(G)**. In the confined cage, iuGC group presented an increase in the number of 22 kHz USVs **(H)** and freezing behavior **(I)**. Upon habituation, iuGC animals no longer presented this anxious behavior (day 2). CONT, control animals; iuGC, *in utero* GC exposed animals; USVs, ultrasonic vocalizations. *p < 0.05, **p < 0.001, and ***p < 0.0001; **(A–C)** *n* = 16; **(D,E)** *n* = 11–13; **(F,G)** *n* = 10, **(H,I)** *n* = 8–16.

In the OF, iuGC animals presented a decrease in the number of ambulatory counts (Figure 1A, *t* = 2.197, *p* = 0.037) and total distance traveled (Figure 1B, *t* = 3.002, *p* = 0.006) when compared with control animals. In addition, iuGC animals exhibited a decrease in the percentage of time spent in the center of the arena (Figure 1C, *t* = 2.416, *p* = 0.023).

In the EPM, iuGC animals spent significantly less time in the open arms (Figure 1D, *t* = 2.947, *p* = 0.009) and presented a reduction in the number of open arms entries (Figure 1E, *t* = 2.375, *p* = 0.031), when compared with control animals. No differences were found in the time and entries in the closed arms (data not shown; time: *t* = 0.479, *p* = 0.637; entries: *t* = 0.872, *p* = 0.392).

In the L/D test, iuGC animals presented an increase in the ratio dark/light (Figure 1F, *t* = 2.765, *p* = 0.014) and no differences in distance traveled in both compartments (Figure 1G, dark: *t* = 0.117, *p* = 0.909; light: *t* = 0.137, *p* = 0.893).

In the confined cage, while control animals rarely emitted aversive 22 kHz USVs, iuGC animals vocalized throughout the exposure (Figure 1H; *U* = 81, *p* = 0.016). Similarly, iuGC group presented increased freezing behavior (Figure 1I; *t* = 2.846, *p* = 0.013). Upon habituation to the cage (2 days of exposure), iuGC animals no longer presented this anxious-like response.

**Figure 2:**
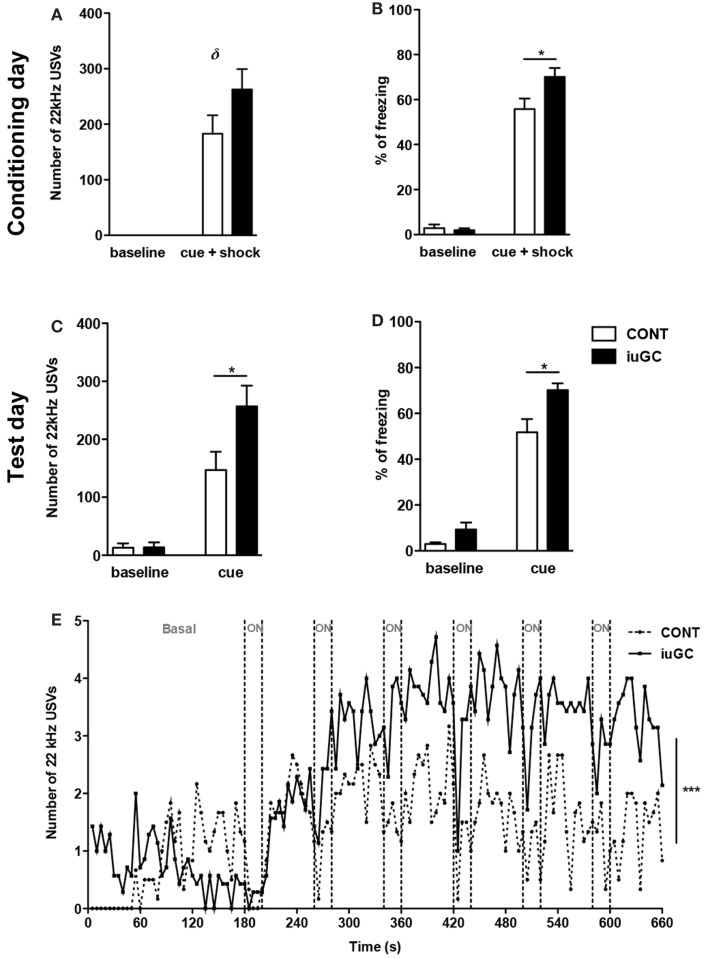
**Prenatal exposure to GC leads to amplified response to adverse stimulus**. **(A)** Number of 22 kHz USVs on the conditioning day (cue + shock) of the fear-conditioning paradigm. iuGC group emitted more negative calls than control animals in the light/shock period. **(B)** Similarly, iuGC group displayed increased percentage of freezing behavior in comparison with control animals. On the test day, animals were exposed six times to cue but no shock was given. iuGC presented an increase in the number of USVs **(C)** and in freezing time **(D)**. **(E)** iuGC animals are hyper-reactive throughout time, since they emit more context-induced 22 kHz vocalizations than control group, especially in the first 60 s. Upon first cue exposure (ON period), both groups increased the number of USVs with no major differences between them. However, upon second cue exposure, iuGC animals emitted more negative USVs than controls and remained over-reactive during time. CONT, control animals; iuGC, *in utero* GC exposed animals; USVs, ultrasonic vocalizations. *p < 0.05, **p < 0.001, ***p < 0.0001, δ: trend, *p* = 0.130; **(A–E)** *n* = 8–16.

To investigate reactivity to an adverse stimulus, we performed a variation of the classical fear-conditioning paradigm. Animals were conditioned to a cue (light) predicting painful electric shocks. After cage habituation, animals were given six pairs of cue-shock. No emission of 22 kHz USVs was observed in the baseline phase (0–3 min), but upon light/shock pairings, as expected, both groups significantly emitted more negative USVs. iuGC animals emitted negative USVs to a greater extent than control animals (Figure 2A, *t* = 1.562, *p* = 0.130) and also presented an increase in freezing behavior time (Figure 2B, *t* = 2.355, *p* = 0.034). After this conditioning session, in the next day, animals were exposed to the cue, but no shock was given. In the initial period, both groups emitted more 22 kHz USVs than the day before (*F*_1,58_ = 4.10, *p* = 0.047); cue exposure elicited more negative USVs in both groups, but again, iuGC animals were over-reactive (Figure 2C, *t* = 2.804, *p* = 0.011). Analysis of freezing behavior further confirmed the phenotype of iuGC group (Figure 2D, *t* = 2.087, *p* = 0.049). Plotting the number of negative USVs along time further confirms that iuGC animals were over-reactive to the cue predicting the adverse stimulus, since iuGC group emitted more context-induced 22 kHz vocalizations than control group, especially in the first 60 s (Figure 2E, *F*_1,131_ = 104.42, *p* < 0.0001). Upon the first cue exposure (ON period), both groups increased the number of USVs with no major differences between them. However, upon second cue exposure, iuGC animals emitted more negative USVs than control group and remained over-reactive throughout. The pattern of 22 kHz USVs emission in both groups is interesting since immediately after the light is turned off, both groups emit more negative vocalizations, indicative of the consolidated association of the cue with the electric shock.

### iuGC exposure induces prominent cholinergic alterations

**Figure 3:**
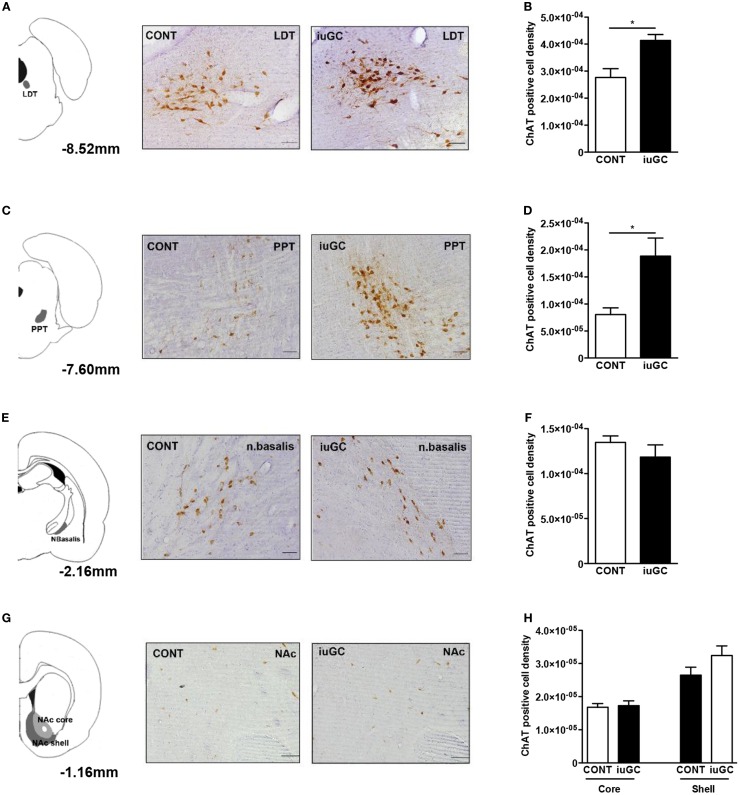
**Mesopontine cholinergic de-regulation in iuGC animals**. **(A)** LDT ChAT immunohistochemistry revealed that iuGC animals presented increased ChAT expression **(B)**. **(C,D)** Similarly, iuGC animals present increased ChAT staining in the PPT region. Conversely, no differences were found in ChAT expression in the nucleus basalis of Meynert **(E,F)** or in the nucleus accumbens core or shell **(G,H)**. Representative images of coronal brain sections; numbers represent distance in mm posterior to bregma. CONT, control animals; iuGC, *in utero* GC exposed animals; LDT, laterodorsal tegmental nucleus; NAc, nucleus accumbens; PPT, pedunculopontine tegmental nucleus. *p < 0.05, **p < 0.001, ***p < 0.0001; **(A)** *n* = 4–7. Scale bars: 50 μm.

Pharmacological studies suggest that the ascending cholinergic tegmental system is responsible for the initiation and production of negative vocalizations in rodents (43–45). In addition, cholinergic signaling is highly responsive to stress/GC (46) and is important for the manifestation of aversive behaviors (47). Considering these findings, we decided to further explore the impact of iuGC exposure in the cholinergic circuitry. To do so, we have performed IHC against ChAT, the key enzyme in ACh synthesis. The number of ChAT-positive cells in the LDT was significantly higher in iuGC animals when compared to control group (Figures 3A,B; 49% increase; *U* = 3, *p* = 0.026). The PPT, a LDT adjacent region, also presented substantial increase in the number of ChAT-positive cells (Figures 3C,D; *U* = 2, *p* = 0.024). On the contrary, the nucleus basalis of Meynert did not show any significant alteration in the number of cholinergic positive cells (Figures 3E,F; *U* = 8, *p* = 0.315). Other regions such as the NAc (core and shell), rich in cholinergic interneurons, also did not show any differences in the number of ChAT-positive cells (Figures 3G,H; core: *U* = 13, *p* = 0.927; shell: *U* = 6, *p* = 0.164).

**Figure 4:**
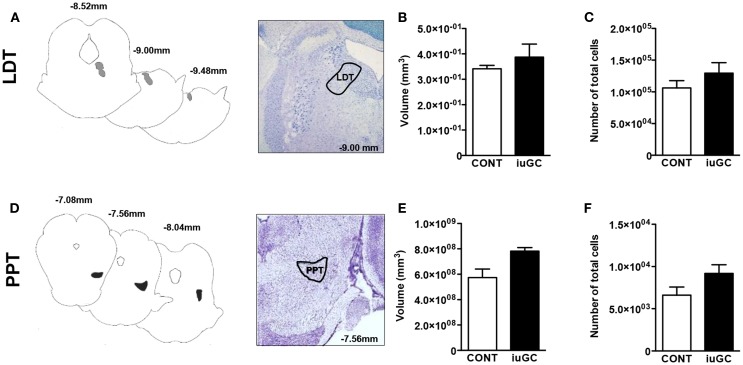
**Stereological data of LDT and PPT regions**. No major differences were observed in the volumes and cell numbers of LDT **(A–C)** and PPT regions **(D–F)** of iuGC animals when compared to controls. Representative images of coronal brain sections; numbers represent distance in millimeters posterior to bregma. CONT, control animals; iuGC, *in utero* GC exposed animals; LDT, laterodorsal tegmental nucleus; PPT, pedunculopontine tegmental nucleus. **(B,C)** *n* = 5–6; **(E,F)** *n* = 4.

Because iuGC can induce relevant structural changes, we measured the volume and number of cells in the LDT and PPT nuclei. No statistical differences regarding the volume of LDT were found between control and iuGC animals (Figures 4A–C; volume: *U* = 5, *p* = 0.486; cell numbers: *U* = 3, *p* = 0.200). In the PPT, no significant differences in volume and cell numbers were found (Figures 4D–F; volume: *U* = 2, *p* = 0.114; cell numbers: *U* = 2, *p* = 0.229).

To assess if the observed increase in ChAT-positive cells in iuGC group was translated into augmented ACh release, we measured the levels of ACh in cholinoceptive regions. A trend for increased ACh levels in the hypothalamus and the amygdala was found (data not shown).

### Cholinergic neurons in the LDT and PPT are differentially activated upon adverse stimulus in iuGC animals

To better determine the impact of iuGC in cholinergic neurons, and the relevance of such changes in reaction to adverse stimuli, we evaluated neuronal activation patterns using c-fos labeling in combination with ChAT after the fear-conditioning protocol.

**Figure 5:**
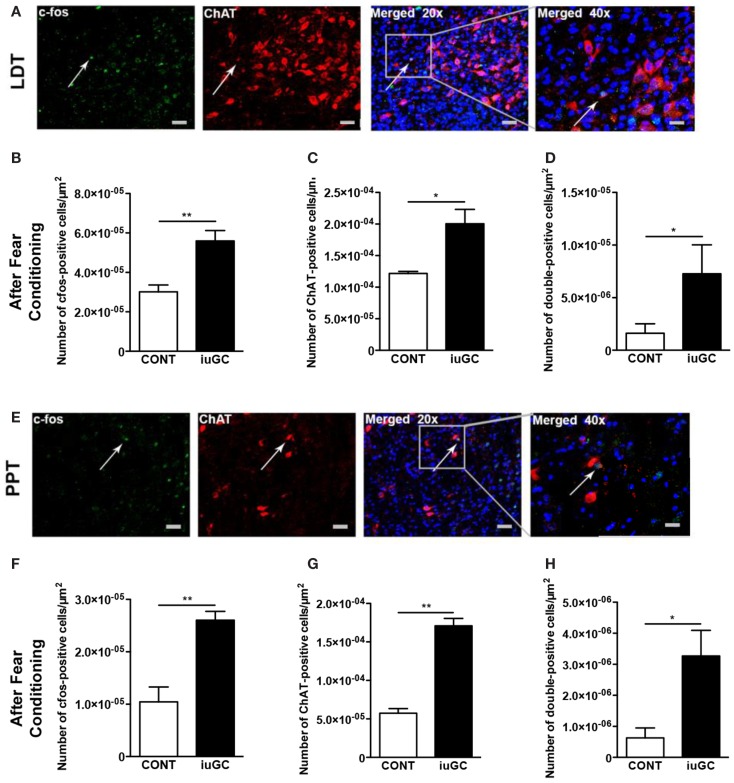
**Differential cholinergic activation of the LDT and PPT regions in iuGC animals after an adverse stimulus**. **(A)** Representative images of LDT ChAT (red labeling) and c-fos (green labeling) immunostaining in animals exposed to the fear-conditioning test. **(B)** Increased c-fos activation in iuGC animals. **(C)** ChAT expression was increased in iuGC animals in comparison to controls. **(D)** The number of c-fos/ChAT-positive cells was substantially increased in the LDT of iuGC animals. **(E)** Representative images of PPT ChAT and c-fos immunostaining in animals exposed to the fear-conditioning test. **(F)** Increased c-fos staining in iuGC animals. **(G)** ChAT expression was augmented in iuGC animals. **(H)** The number of c-fos/ChAT-positive cells is significantly increased in the PPT of iuGC group. CONT, control animals; iuGC, *in utero* GC exposed animals; LDT, laterodorsal tegmental nucleus; PPT, pedunculopontine tegmental nucleus. *p < 0.05, **p < 0.001; **(A–H)** *n* = 6. Scale bars: 50 μm.

Briefly, animals were subjected to the modified fear-conditioning protocol, and sacrificed 90 min after stimuli on the test day. As depicted in Figure 5, after an adverse stimulus, iuGC animals presented a significant increase (85%) in the number of c-fos-positive cells in the LDT (Figures 5A,B; *U* = 0, *p* = 0.008). The number of ChAT-positive cells was also substantially augmented (64%) in iuGC animals when compared to control animals (Figure 5C; *U* = 0, *p* = 0.014).

Regarding the PPT, after the adverse stimuli, iuGC animals present 2.5 times more c-fos-positive cells than control animals (Figures 5E,F; *U* = 0, *p* = 0.002). Similarly, the number of ChAT-positive cells was also increased in iuGC animals (Figure 5G; *U* = 0, *p* = 0.002).

To determine if the observed increase in c-fos-positive cells was due to enhanced cholinergic activation, we also quantified the number of c-fos/ChAT-positive cells after fear conditioning. Remarkably, iuGC animals presented a substantial increase (186%) in the number of c-fos/ChAT-positive cells in the LDT in comparison to control animals (Figure 5D; *U* = 4, *p* = 0.029). Similarly, in the PPT, we observed that iuGC animals display a four-time increase in the number of double c-fos/ChAT-positive cells (Figure 5H; 3.358 vs. 0.801%, *U* = 4.5, *p* = 0.030).

## Discussion

Confirming previous findings (12, 14, 48), we observed that iuGC animals present an anxious phenotype. In the EPM, iuGC animals presented a decrease in the time and entries in the open arms when compared with control animals, in accordance with the L/D test, where they spent less time in the anxiogenic compartment. Moreover, iuGC animals emitted more 22 kHz negative calls and presented enhanced freezing behavior in the confined cage paradigm. These differences were eliminated by habituation. This is in accordance with previous work showing that rats that are highly anxious tend to vocalize more often and present augmented freezing time during aversive stimuli than rats that display low anxiety-like trait (35). In the fear-conditioning paradigm, iuGC animals emitted more negative calls and enhanced freezing behavior on the conditioning day. This suggests an emotional over-reactivity of iuGC animals to adverse stimulus. In further support of this idea, iuGC animals emitted more negative calls than control subjects in response to the cue predicting the harmful stimulus and during ISI, suggesting an over-reactive response. Altogether, our data confirms that iuGC exposure leads to anxious behavior and exacerbated response to stressful events in adulthood, a finding also observed in other stress models (49–51). Similarly, a rat line that displays signs of extreme trait anxiety also presents increased stress vulnerability and reactivity (52).

One remarkable finding was the increased emission of negative calls in iuGC animals, hinting differential activity of the mesopontine cholinergic circuitry, that mainly comprises projections from the LDT region (43, 44, 53, 54). In accordance, we found increased ChAT expression in the LDT of iuGC animals in a basal situation, suggesting overproduction of ACh. Upon exposure to a cue predicting an adverse stimulus, there was a marked increase in the number of c-fos/ChAT-positive cells, potentially explaining iuGC behavioral response. Indeed it was shown that LDT activation induces a complex state of defensiveness, critical for alarm responses to dangerous stimuli (44, 45). Our results are in accordance with previous work, in which they have shown that pharmacological stimulation of the LDT lead to an increase in the emission of 22 kHz USVs (44). In addition, the increased LDT cholinergic signaling could also be implicated in the impaired negative HPA axis feedback of iuGC animals (12) since LDT-arising ACh enhances ACTH and CRF release (28, 55, 56). On the other hand, GC also modulate cholinergic signaling (19), suggesting a reciprocal ACh-GC control that may culminate in a vicious loop.

Alike the LDT, iuGC animals presented increased PPT cholinergic signaling both in a basal situation and after exposure to the cue predicting an adverse stimulus. Apart from the classical role of PPT in sleep and arousal, lesion studies suggest a role in anxiety modulation, although both anxiogenic and anxiolytic effects were found depending on the degree and type of lesion. Excitotoxic and electrolytic PPT lesions induce an anxiogenic-like status (57, 58), contrary to one study that suggests a slight anxiolytic effect (59). On the other hand, ibotenic lesions seem to reduce anxious-like phenotype (60). Apart from distinct technical procedures, it has been suggested that opposing results may arise from damage in different sub-regions within the PPT (pars compacta vs. dissipata) or in surrounding regions, namely the cuneiform nucleus (59, 61).

This programing effect of iuGC exposure is not so surprising considering the importance of GC receptors for the maturation of medial septal and hippocampal cholinergic neurons (62, 63), yet, more studies need to be performed to understand how GC exert long-lasting functional and molecular changes in these neurons.

*In utero* glucocorticoid exposure-induced alterations in the mesopontine cholinergic pathway may go beyond a direct effect of cholinergic inputs on behavioral output. For instance, LDT-dependent cholinergic activation of VTA evokes dopamine release in the NAc (20, 21, 64) driving motivational and reward behaviors (65, 66). However, these findings are somewhat contradictory to the VTA-accumbal hypodopaminergia and anhedonia observed in iuGC animals (6, 14, 15). One possible explanation is that the sustained augmented LDT-VTA ACh signaling could desensitize or alter the expression/epigenetic status of the nicotinic/muscarinic receptors as a compensatory mechanism. Indeed, GC or acute stress can induce prominent ACh release in specific brain regions (46, 67, 68), and transiently change the expression levels of different cholinergic players through c-fos binding to promoter regions of target genes in order to maintain the homeostasis (19). Additional studies focusing on the regulation of the expression of different cholinergic players by GC will be critical to better comprehend our findings.

In summary, our results show for the first time that prenatal GC exposure programs the mesopontine cholinergic pathway, leading to cholinergic hyperactivation of both the LDT and PPT, which in turn can underlie the anxious behavior and enhanced stress reactivity observed in these animals.

## Conflict of Interest Statement

The authors declare that the research was conducted in the absence of any commercial or financial relationships that could be construed as a potential conflict of interest.
